# Ivabradine, the pure heart rate-lowering drug, shown to reduce morbidity and mortality in heart failure

**Published:** 2010

## Introduction

The results of the SHI_f_T study, announced this week at the European Society of Cardiology (ESC) 2010 congress in Stockholm, are likely to change the clinical treatment of chronic heart failure as they provide the first positive results for many years in this difficult field of treatment. The great interest in the outcome of this study was evident from the large number of delegates attending this hot-line session on the first day of the congress.

SHIfT has now shown for the first time that treatment with ivabradine, added to close-to-optimal guideline-directed therapies, was able to further reduce the risk of cardiovascular death and hospitalisation from worsening heart failure in patients with moderate to severe heart failure (LVEF ≤ 35%) and a raised heart rate (above 70 beats per minute).^1^ Ivabradine is a specific inhibitor of the I_f_ current in the sino-atrial node.

The significant relative risk reduction (RRR) of 18% in this primary composite outcome in patients receiving ivabradine therapy was primarily the consequence of the 26% reduction in hospital admissions; 16% of patients taking ivabradine were admitted to hospital with worsening heart failure compared to 21% in the placebo group. The absolute risk reduction achieved was 4.2%. This means that 26 patients would need to be treated for one year to prevent one cardiovascular death or one hospital admission for heart failure.

**Fig. 1.**
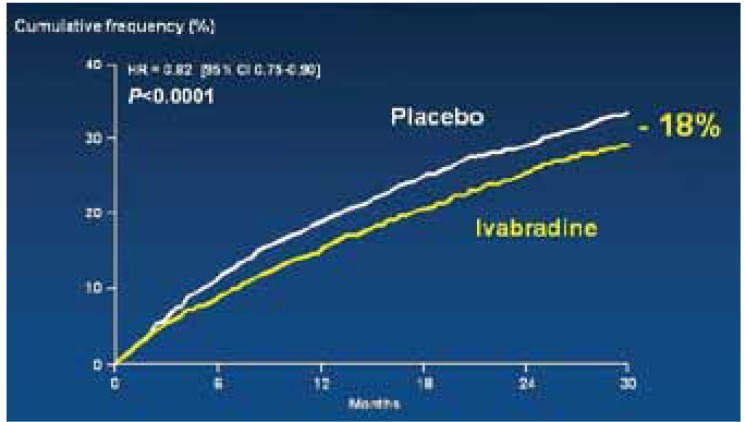
Cardiovascular mortality/heart failure hospitalisation

**Fig. 2.**
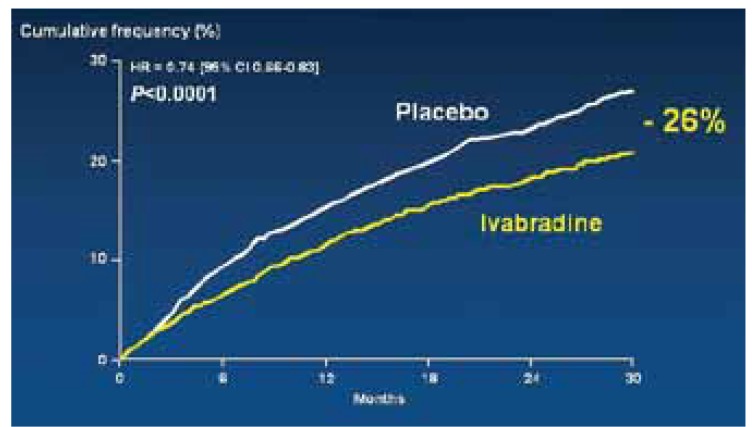
Hospitalisation for heart failure.

Importantly, the benefit of ivabradine therapy was seen early on in the first three months of therapy (Figs 1, 2 ). The other component of the composite endpoint, cardiovascular death, was reduced by 9%, which was not statistically significant.

The 10% reduction in all-cause mortality achieved in the ivabradine arm (16%) compared to 17% in the placebo arm did not reach statistical significance. There were no differences in the incidence of sudden death between the two treatment arms. Heart failure-related deaths were however significantly reduced from 5% in the placebo arm to 3% in the ivabradine arm (RRR: 26%).

This SHI_f_T study of systolic heart failure treatment with the If current inhibitor ivabradine was undertaken to investigate whether lowering heart rate with ivabradine could reduce cardiovascular deaths and hospital admissions from worsening heart failure among patients with chronic heart failure, systolic dysfunction, normal sinus rhythm and an elevated heart rate.

The study included 6 505 patients and was conducted over a median follow-up period of 22.9 months. Patients were mainly men, with an average age of 60 years, and in NYHA classes II and III. Duration of heart failure was three years and patients had to have experienced a hospitalisation event in the 12 months prior to entering the study. Heart failure was mainly of ischaemic origin with 32% of patients (2 086) in both the ivabradine and placebo arms being categorised as having heart failure of non-ischaemic origin.

Presenting the results, Prof Michel Komajda, professor of Cardiology, Université Pierre et Marie Curie, Paris, stressed that the investigators from 37 countries, which excluded the United States of America, where ivabradine is not registered, and Africa, had been encouraged to prescribe the best current standard of care as recommended by the ESC guidelines. These guidelines also form the basis of the South African heart failure treatment guidelines.

The excellent background standard of care was evident at randomisation, with 90% of patients receiving beta-blocker therapy, 93% on ACE inhibitors/angiotensin receptor blocker (ARB) therapy, 84% on diuretics and 60% receiving antialdosterone agents. Device usage was low as per protocol (3%).

Patients entering the study were given a starting dose of 5 mg ivabradine twice daily, which was up-titrated or lowered, depending on the heart rate response. Heart rate was measured by ECG at regular four-monthly intervals throughout the study.

‘The results of SHIfT are vital as there is still a clear unmet clinical need in the treatment of heart failure, which, despite advances in therapy with five drug classes which form the basis of recommended therapy, 50% of patients still die during the first four years. In addition, quality oflife can be very poor; commonly 25% of patients are re-hospitalised within three months after their first admission for heart failure-related complications’, Prof Komajda concluded.
